# Highly sensitive and specific detection of aminoglycoside resistance methylase gene *armA* by multiple cross displacement amplification-CRISPR/Cas12a technique

**DOI:** 10.3389/fcimb.2026.1947114

**Published:** 2026-10-28

**Authors:** Lin Gong, Juan Wang, Yuandong Zhang, Huiqiong Xu, Xiaoli Liu, Xiaomin Chen, Meiling Li, Yao Li, Liqun Wu, Yimei Wang, Xiantao Shen

**Affiliations:** 1 State Key Laboratory of Environment Health (Incubation), Key Laboratory of Environment and Health, Ministry of Education, Key Laboratory of Health Effects of Environmental Pollution, Ministry of Ecology and Environment, School of Public Health, Tongji Medical College, Huazhong University of Science and Technology, Wuhan, Hubei, China; 2 Department of Disinfection and Pest Control, Wuhan Center for Disease Control & Prevention, Wuhan, Hubei, China; 3 Department of Infectious Disease Prevention and Control, Dongxihu District Center for Disease Control and Prevention, Wuhan, Hubei, China; 4 Public Health Laboratory and Testing Center, Wuhan Center for Disease Control & Prevention, Wuhan, Hubei, China

**Keywords:** aminoglycoside resistance, *armA*, CRISPR/Cas12a, detection, MCDA

## Abstract

**Introduction:**

The 16S rRNA methylase *armA* serves as a key molecular determinant conferring high-level resistance to aminoglycoside antibiotics in bacterial pathogens. Development of a rapid and reliable *armA* gene detection assay will improve surveillance of enzyme-producing strains and support drug resistance control in diverse ecological settings.

**Methods:**

A multiple cross displacement amplification (MCDA) combined with CRISPR/Cas12a system assay was established to identify *armA* gene with rapidity, specificity, and sensitivity.

**Results:**

The entire experimental process, including genomic DNA extraction (15 minutes), MCDA reaction (40 minutes), CRISPR/Cas12a-mediated cleavage (5 minutes), and fluorescence detection (10 minutes), can be completed within 70 minutes. In terms of analytical specificity, the assay yielded positive results for all armA-positive isolates and negative results for all non-*armA* samples. The sensitivity of the MCDA-CRISPR/Cas12a assay reached a detection limit as low as 10 copies/mL of armA-positive plasmid DNA. In 129 cultured clinical isolates validated by whole-genome sequencing, the assay achieved 100% concordance for armA detection.

**Discussion:**

The technique established in this study provides a rapid and accurate diagnostic approach for laboratory-based surveillance of armA in clinical and livestock settings, with future potential for point-of-care adaptation following further simplification and validation.

## Introduction

Aminoglycosides antibiotics are widely used for the treatment of clinical infections, especially those caused by Gram-negative bacteria (McDermott et al., 2022). However, due to this widespread application, the emergence of resistance has been a matter of public concern of aminoglycosides, greatly limiting the choice of the therapeutic options. Bacterial resistance to aminoglycosides can occur in a number of ways but most concerning is the production of 16S rRNA methyltransferases that prevents the binding of aminoglycosides to the 30S subunit, mediating high-level resistance to most aminoglycoside antibiotics (Doi and Arakawa, 2007; Vidučić et al., 2025).To date, ten different 16S rRNA methylase genes have been identified, including *armA, rmtA, rmtB, rmtC, rmtD, rmtE, rmtF, rmtG, rmtH* and *npmA (*Galimand et al., 2012). *ArmA* gene is the most common among *Enterobacteriaceae*, which was initially found in *Klebsiella pneumoniae* isolated from patients in 2003 (Yokoyama et al., 2003). Afterwards, this gene was reported in Spain, Japan, Bulgaria, South Korea and China (including Taiwan), showing a trend of wide spread on a global scale (Yan et al., 2004; González-Zorn et al., 2005; Yamane et al., 2005; Wachino et al., 2006; Doi et al., 2007). Meanwhile, *armA* gene has been found in almost all species and genera of Gram-negative bacteria with great clinical significance, including *Enterobacter, Providencia, Klebsiella, Serratia, Shigella, Citrobacter* and none fermentstrains such as *Acinetobacter* and *Pseudomonas (*Yamane et al., 2005; Yamane et al., 2007). Currently, several techniques have been available for the detection of *armA*, such as GeXP analyzer-based multiplex PCR assay, and targeted liquid chromatography-tandem mass spectrometry (LC-MS/MS) (Hu et al., 2013; Foudraine et al., 2021). Nonetheless, these techniques are time-consuming and limited by detection conditions, which are not conducive to rapid pathogen detection. Therefore, a rapid, sensitive, and specific technology for *armA* detection is imperative to control the abuse of antibiotics.

The CRISPR/Cas system, which is well-known adaptive immunity system, is widespread in archaea and bacteria (Ishino et al., 2018). Due to its characteristics of high sensitivity and specificity, it has been widely applied in the fields of gene editing and nucleic acid test (Doudna and Charpentier, 2014; Aman et al., 2020; Bhatia et al., 2023). Among the wide variety of Cas endonucleases, Cas12a, guided by CRISPR RNA (crRNA), specifically recognizes and cleaves double‐stranded DNA (dsDNA) targets bearing the TTTN Protospacer Adjacent Motif (PAM). Formation of the crRNA‐Cas12a‐target complex triggers its trans‐cleavage activity, which non‐specifically cuts nearby single‐stranded DNA (ssDNA) reporters to generate detectable signals (Chen et al., 2018; Li et al., 2021).

Generally, the CRISPR/Cas system can be used in conjunction with nucleic acid amplification techniques to effectively enhance detection sensitivity (Zeng et al., 2024). Recombinase polymerase amplification (RPA) coupled with CRISPR/Cas12a represents a popular isothermal strategy and has shown promising performance for rapid pathogen testing, including recent reports for *Mycoplasma pneumoniae* clinical detection (Li et al., 2022). Nevertheless, RPA relies on a multi-protein reaction cocktail (recombinase, single-strand-binding protein, and polymerase), requires separate addition of the magnesium acetate activator, and is susceptible to non-specific amplification, which complicates assay setup and result interpretation (Craw and Balachandran, 2012). Since its development in 2015, multiple cross displacement amplification (MCDA) has been established as an effective isothermal amplification technique owing to its high sensitivity and specificity (Wang et al., 2015). MCDA depends only on Bst DNA polymerase and employs ten primers that recognize ten distinct target sequence regions, conferring intrinsic advantages in target specificity and reagent compatibility compared with RPA (Mao et al., 2025; Yuan et al., 2026). So far, MCDA has been successfully employed in the detection of diverse pathogens, including bacteria and viruses (Li et al., 2020; Luu et al., 2021; Hu et al., 2025). Given its rapid turnaround time and minimal equipment requirements, MCDA technology is highly suitable for point-of-care detection (Chen et al., 2021). Currently, only a limited number of studies have reported the use of MCDA in conjunction with the CRISPR/Cas12a system for the detection of pathogens such as *Coxsackievirus* A16, *Mycobacterium tuberculosis*, and *Haemophilus influenzae (*Jia et al., 2023; Jia et al., 2024; Liang et al., 2026). Moreover, no analogous method has yet been established for the detection of antimicrobial resistance.

In this study, we proposed integrating the MCDA with the CRISPR/Cas12a system to establish a novel method for the rapid detection of the *armA* gene (MCDA-CRISPR/Cas12a). This approach holds significant promise for clinical detection of the *armA* gene, thereby offering a valuable tool for early diagnosis and antimicrobial resistance surveillance.

## Materials and methods

### Materials and instruments

22 *armA*-positive and 24 *armA-*negative isolates were collected in this study (**Supplementary Table S1**). The *armA*-producing bacterial organisms included *Escherichia coli, Klebsiella pneumoniae, Pseudomonas aeruginosa* and *Enterobacter cloacae*, 8 kinds of resistance genes consisted of *blaKPC-2*, *rmtB*, *blaTEM-1B*, *blaNDM-1*, *blaIMP-4*, *blaOXA-1*, *qnrS1*, and *sul1* were incorporated in the *armA*-negative strains. All of the resistance genes above were verified using PCR and sequencing. The DNA extraction kits were purchased from Kangwei Century Biotechnology Co., Ltd. (Beijing, China). The *armA* and *rmtA* plasmids were synthesized by Qingke Biotechnology Co., Ltd. (Beijing, China). pUC57 served as the vector, and competent cells derived from the *Escherichia coli* TOP10 strain were used for transformation. The MCDA reaction was carried out with a DNA isothermal amplification kit, which was composed of reaction buffer and Bst DNA polymerase (HuiDeXin Biotechnology Development Co., Ltd., Tianjin, China). The isothermal condition of MCDA reaction was supplied by a heater (MTH-100, MiU Instruments Co., Ltd., Hangzhou, China). Cas12a nuclease were obtained from Magigen Biotechnology Co., Ltd. (Guangzhou, China). At last, the obtained reaction products could be detected in real-time PCR (LightCycler 480, Roche Diagnostics, Basel, Switzerland).

### MCDA primers and crRNA preparation

A total of 10 primers targeting the *armA* gene (GenBank accession number KU984333.1) were designed using Primer Premier 6.0 software based on the principle of MCDA amplification (Wang et al., 2016). The design criteria were as follows: a length of 18–25 nucleotides (with an upper limit of 30 nt), a GC content of 45–60%, a melting temperature (Tm) of 60–65 °C, the absence of adenine (A) at the 3’ terminus, and no significant hairpin, self-dimer, or cross-dimer formation. The primer set comprised two cross primers (CP1 and CP2), two displacement primers (F1 and F2), and six amplification primers (C1, C2, R1, R2, D1, and D2). Primer specificity was subsequently analyzed and validated using BLAST software. In parallel, crRNA for the CRISPR/Cas12a system and the corresponding detection probe were designed according to the target product sequence. To further support primer and crRNA specificity bioinformatically, we have performed BLAST analysis to show the comparison of *armA* with the closest non-*armA* sequence and other 16S rRNA methylase genes. All primers, crRNA, and probe employed in this study were synthesized by Qingke Biotechnology Co., Ltd. (Beijing, China). The detailed sequences and schematic representation of the MCDA primers and probe are provided in **Figure 1B**; **Table 1**.

**Figure 1:**
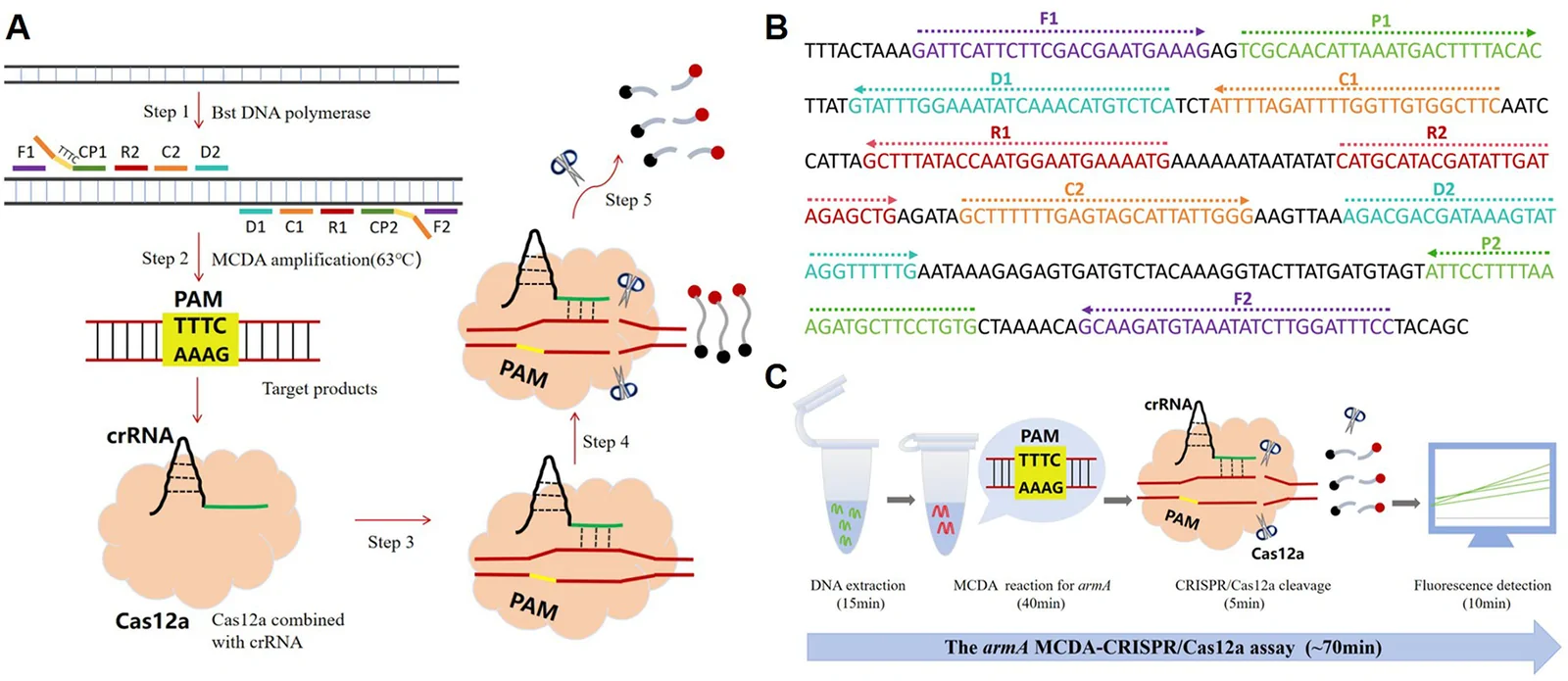
Schematic diagram of the *armA* MCDA-CRISPR/Cas12a technique. **(A)** Outline of the MCDA-CRISPR/Cas12a assay for *armA* detection. Target sequence containing a PAM (TTTC) site is specifically amplified by the MCDA reaction (steps 1 to 2). When combining with amplicons products, the crRNA-CRISPR/Cas12a complex is induced to nonspecifically cleave the ssDNA (both ends labeled with fluorophores) (steps 3 to 5). **(B)** The location of the five primers pairs on the partial *armA* gene sequence. Different pairs of primers were represented by various of colors. The arrowed lines showed the amplification direction of primers. **(C)** Schematic of the *armA* MCDA-CRISPR/Cas12a assay workflow. The entire workflow of the technique includes 4 steps: DNA extraction, MCDA reaction, CRISPR/Cas12a-mediated cleavage, and fluorescence detection.

**Table 1:** PCR primers for detecting the *armA* gene, crRNA, probe and primers of the MCDA-CRISPR/Cas12a technology.

Name	Serial number	Sequences and modifications (5′ -3′)	Length^a^
Primer-PCR-F	SEQ ID NO.1	ATGGATAAGAATGATGTT	18 nt
Primer-PCR-R	SEQ ID NO.2	TTATTTCTGAAATCCACT	18 nt
crRNA^b^	SEQ ID NO.3	UAAUUUCUACUGUUGUAGAUUCGCAACAUUAAAUGACUUUUACAC	45 nt
Probe^c^	SEQ ID NO.4	FAM-TATTAT-BHQ1	6 nt
Primer-F1	SEQ ID NO.5	GATTCATTCTTCGACGAATGAAAG	24 nt
Primer-F2	SEQ ID NO.6	GGAAATCCAAGATATTTACATCTTGC	26 nt
Primer-CP1	SEQ ID NO.7	GAAGCCACAACCAAAATCTAAAATTTTCTCGCAACATTAAATGACTTTTACAC	53 nt
Primer-CP2	SEQ ID NO.8	GCTTTTTTGAGTAGCATTATTGGGCACAGGAAGCATCTTTAAAAGGAAT	49 nt
Primer-C1	SEQ ID NO.9	GAAGCCACAACCAAAATCTAAAAT	24 nt
Primer-C2	SEQ ID NO.10	GCTTTTTTGAGTAGCATTATTGGG	24 nt
Primer-D1	SEQ ID NO.11	TGAGACATGTTTGATATTTCCAAATAC	27 nt
Primer-D2	SEQ ID NO.12	AGACGACGATAAAGTATAGGTTTTTG	26 nt
Primer-R1	SEQ ID NO.13	CATTTTCATTCCATTGGTATAAAGC	25 nt
Primer-R2	SEQ ID NO.14	CATGCATACGATATTGATAGAGCTG	25 nt

a nt meant the nucleotide.

b The crRNA comprised a 25-nt Cas12a scaffold (UAAUUUCUACUGUUGUAGAU) and a 20-nt *armA*-specific spacer (UUCCGACAUCUAAAUGACUU).

c The probe was 5’-FAM and 3’-BHQ1 labeled.

### MCDA amplification

The MCDA amplification was performed in a 24.2 µL reaction system. The reaction mixture included 12.5 µL 2 × reaction buffer, 1 μL Bst DNA polymerase, 1 μL indicator (malachite green), 0.4 µL cross primers CP1 and CP2 (1.6 µM each), 0.1 µL displacement primers F1 and F2 (0.4 µM each), 0.2 µL amplification primers C1, C2, R1, R2, D1, and D2 (0.8 µM each), and 1 µL DNA template, with deionized water added to a final volume of 24.2 µL. The reaction was carried out at 63 °C for 40 min. *Klebsiella pneumoniae* producing *blaNDM-1* gene (WHCDC-FY57) and deionized water were used as negative control and blank control respectively. To obtain a better reaction temperature, the MCDA reaction was executed in the range of 60 to 67 °C with 1 °C intervals.

### CRISPR/Cas12a detection

The CRISPR/Cas12a detection was accomplished in a 50 µL reaction system. The reaction system (50 µL) consisted of 5 µL 10× reaction buffer (500 mM NaCl, 100 mM Tris-Tricine [pH 7.9], 100 mM MgCl_2_, and 10 mM DTT), 0.5 µL crRNA (1 µM), 0.5 µL ssDNA probe (0.5 µM), 0.5 µL Cas12a protein (20 nM), 2 µL MCDA product, and 41.5 µL deionized water. Then, the reaction temperature was conducted at 37 °C for 5 minutes, followed by real-time fluorescence detector used to analyze the fluorescent signal.

### The sensitivity and specificity of MCDA-CRISPR/Cas12a technique

To evaluate the Limit of Detection (LoD) of MCDA-CRISPR/Cas12a assay, serial dilutions of *armA* plasmid DNA were prepared to cover a range from 1.05 × 10^4^ copies/µL to 1 copy/µL (1.05×10^4^, 1.05×10^3^, 1.05×10^2^, 50, 10, 5, 1). According to the previous detection system, negative control and blank control were set up simultaneously. Following MCDA amplification, the resulting products were analyzed using three parallel detection methods: colorimetric indication, conventional agarose gel electrophoresis and CRISPR/Cas12a-based detection. The sensitivity test was repeated 7 times using fluorescence detection. The specificity of MCDA-CRISPR/Cas12a method was evaluated using 46 strains of bacteria (22 *armA*-positive and 24 non-*armA* isolates) and a plasmid carrying the *rmtA* gene. Real-time fluorescence was used to validate the result. The specificity test was repeated 5 times.

### Clinical sample verification

From 2018 to 2024, a total of 129 clinical samples of carbapenemase-producing Gram-negative bacteria were collected from eight hospitals in Wuhan. These samples were primarily derived from blood, sputum, urine, and bronchoalveolar lavage fluid. The samples were subsequently sent to Novogene Co., Ltd. (Beijing, China), where a 2 × 350 bp DNA library was constructed. Whole-genome sequencing (WGS) was performed using the Illumina PE150 high-throughput sequencer (Illumina, Inc., California, USA). The filtered and assembled sequencing data were submitted to the Center for Genomic Epidemiology (CGE) database (http://www.genomicepidemiology.org/services) for resistance genes comparison. Finally, the performance of the MCDA-CRISPR/Cas12a method was evaluated by comparing its results with those obtained from whole-genome sequencing. The relevant information of 129 bacterial strains was displayed in **Supplementary Table S2**.

### The sensitivity of PCR assay

Serial dilutions of *armA* plasmid DNA ranging from 1.05×10^8^ copies/μL to 1 copy/μL(1.05×10^8^, 1.05×10^7^, 1.05×10^6^, 1.05×10^5^, 1.05×10^4^, 1.05×10^3^, 1.05×10^2^, 50, 10, 5, 1) were used as templates to determine the limit of detection of conventional PCR assay. The primers used for PCR assay are listed in **Table 1**.

## Results

### Confirmation test of the MCDA-CRISPR/Cas12a technique

As depicted in **Figure 1A**, the mechanism of MCDA-CRISPR/Cas12a system mainly consists of the following two key processes: MCDA pre-amplification and CRISPR/Cas12a detection. In the MCDA assay, the target sequence containing a PAM site (TTTC) can be amplified specifically and rapidly within 40 minutes at a constant temperature of 63 °C. Specifically, the MCDA reaction employs a set of ten primers that collectively recognize ten distinct regions within the target DNA sequence. This primer set comprises 2 displacement primers (F1 and F2), 2 cross primers (CP1 and CP2), and 6 amplification primers (D1, D2, C1, C2, R1, and R2). During strand invasion, displacement primers F1 and F2 displace the annealed crossover primers CP1 and CP2, thereby promoting template switching and enhancing reaction processivity. Working synergistically under the catalytic activity of Bst DNA polymerase, the six amplification primers drive rapid isothermal DNA synthesis, significantly accelerating amplification kinetics and improving amplicon yield. Collectively, the polymerase-primer complex formed by the combination of Bst DNA polymerase and specific primer enables highly efficient, specific, and robust amplification of the target gene.

During the detection stage, the crRNA-CRISPR/Cas12a complex recognizes the target sequence via the PAM site, thereby successfully activating the Cas12a effector. Once activated, Cas12a cleaves the target substrate and simultaneously performs non-specific *trans*-cleavage of fluorophore-labeled ssDNA probes within the reaction system. This process releases fluorescent signals, which can be collected as indicators for detection results. The entire diagnostic procedure of the MCDA-CRISPR/Cas12a assay—including genomic DNA extraction (15 minutes), MCDA reaction (40 minutes), CRISPR/Cas12a-mediated cleavage (5 minutes), and fluorescence detection (10 minutes)—can be completed within 70 minutes (**Figure 1C**).

To validate the feasibility of MCDA-CRISPR/Cas12a assay, the MCDA pre-amplification was performed at 63 °C for 40 min. Then, three monitoring methods were used to verify MCDA pre-amplification results, including colorimetric indication, agarose gel electrophoresis and CRISPR/Cas12a detection. 5 μL of each product was loaded on the 1.5% agarose gel, and the results of agarose gel electrophoresis showed that positive reaction appeared in the tube loaded with 1.05×10^5^ copies/μL of *armA*-positive plasmid DNA, but not with *Klebsiella pneumoniae* producing *blaNDM-1* gene (WHCDC-FY57), and deionized water. The same results were obtained using colorimetric indication and fluorescence detection in the following CRISPR/Cas12a assay (**Figure 2**). Therefore, these data indicated that the primers and crRNA were effective in the experiment. Furthermore, to investigate the conservation of the *armA* gene sequence, we retrieved *armA* gene sequences from seven different bacterial species harboring the *armA* gene from NCBI and performed sequence alignment of the regions targeted by the primers and crRNA. The results showed that these target regions are conserved across the analyzed strains (**Supplementary Figure S1**). This further demonstrates the robustness of the established method.

**Figure 2:**
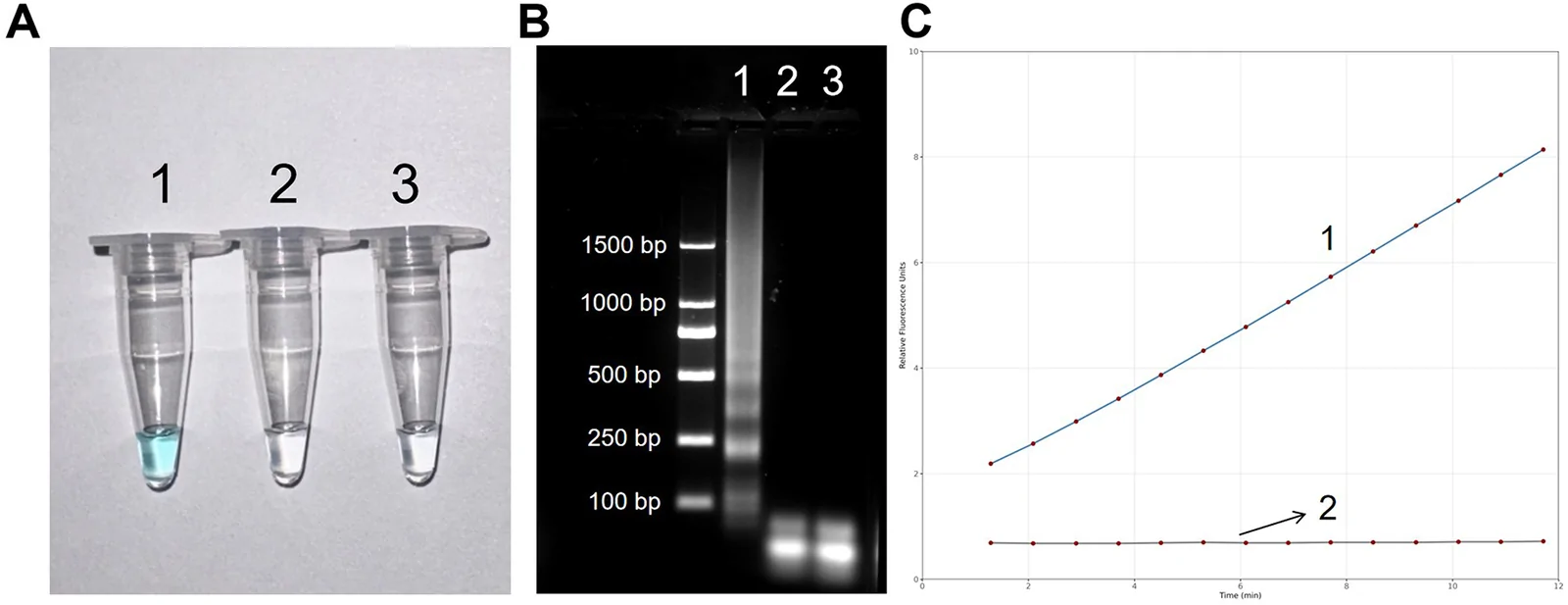
Confirmation of the *armA* MCDA-CRISPR/Cas12a assay. **(A)** MCDA products; **(B)** gel electrophoresis analysis of MCDA products; **(C)** fluorescence detection. 1–3 corresponded to 1.05 × 10^5^ copies/µL of *armA*-positive plasmid DNA, the negative control (WHCDC-FY57, a *blaNDM-1*-producing *Klebsiella pneumoniae* strain), and the blank control (deionized water), respectively. Line 3 also represented a negative signal and was therefore omitted from the **(C)**.

### Optimal reaction condition for MCDA-CRISPR/Cas12a assay

The reaction conditions for MCDA amplification and CRISPR/Cas12a detection were primarily referenced from previous studies (Gong et al., 2019; Gong et al., 2023). To ensure the high efficiency of MCDA augment, the optimal reaction temperature was evaluated. The reaction systems were performed at eight different temperatures ranging from 60 to 67 °C with 1 °C increments, using *armA*-positive plasmid DNA with a concentration of 1.05×10^5^ copies/μL. Agarose gel electrophoresis results showed that 61 to 67 °C were the better reaction temperature of MCDA amplification (**Supplementary Figure S2**). Thus, 63 °C was chosen as the optimal reaction temperature and applied to the following MCDA-CRISPR/Cas12a assay.

### Sensitivity and specificity estimation of MCDA-CRISPR/Cas12a technique

Serial dilutions of the *armA*-positive plasmid DNA were used as templates to evaluate the detection limit of MCDA-CRISPR/Cas12a assay. The results were observed by three methods of colorimetric indication, agarose gel electrophoresis analysis and fluorescence signals detection.

The detection rate, reproducibility, and signal-to-noise ratio (SNR) data of the assay at different plasmid DNA concentrations are shown in **Supplementary Table S3**. Probit regression analysis yielded a 95% detection limit of 7.2 copies/μL (95% CI: 5.8–9.5 copies/μL). Considering practical applicability, the LoD was conservatively reported as 10 copies/μL. The intra-assay CV (Coefficient of Variation) at the LoD concentration (10 copies/μL) was 21.99% (n=7). The relatively high CV at various template concentrations may be attributable to Poisson distribution effects and the uncontrollable nature of Cas12a-mediated indiscriminate cleavage of ssDNA probes, which is common for ultrasensitive CRISPR-based nucleic acid detection assays. The SNR at the LoD (10 copies/μL) was 72.83, calculated as (mean positive signal - mean no-template control [NTC] signal) divided by the standard deviation (SD) of the NTC, indicating a robust and clearly distinguishable positive signal. Above all, as shown in **Figure 3**, the LoD of MCDA-CRISPR/Cas12a assay was 10 copies/μL of *armA*-positive plasmid DNA, and the results of the three detection methods were consistent. Representative kinetic fluorescence curves were shown in **Supplementary Figure S3**. Fluorescence curves for all replicates at the LoD (10 copies/μL) was shown in **Supplementary Figure S4**.

**Figure 3:**
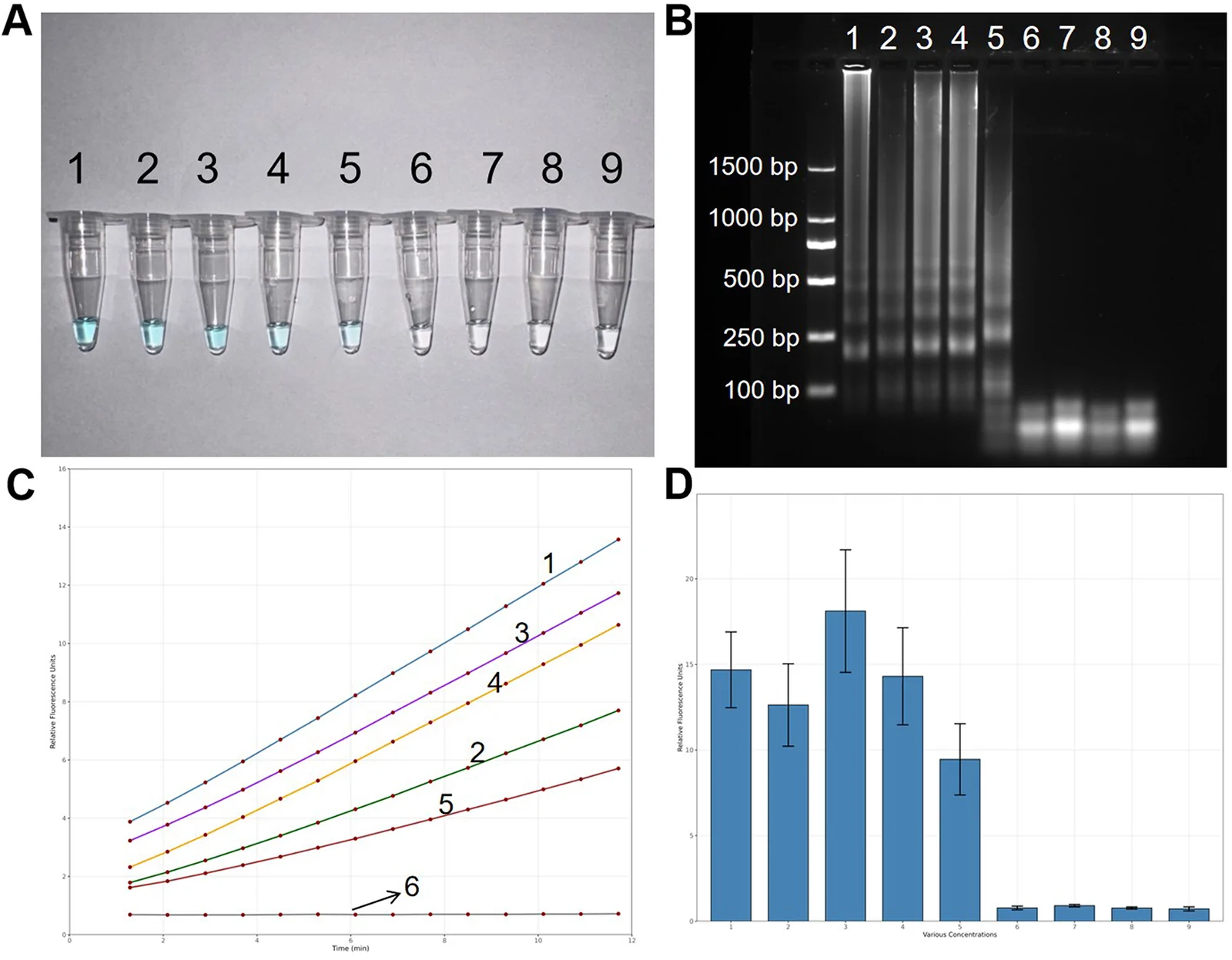
Sensitivity of the *armA* MCDA-CRISPR/Cas12a assay. **(A–C)** Detection results obtained by three different methods: colorimetric indication **(A)**, gel electrophoresis **(B)**, and fluorescence detection **(C)**. **(D)** Statistical analysis of fluorescence intensity from seven replicate experiments. Lanes 1–9 corresponded to 1.05×10^4^ copies/μL, 1.05×10^3^ copies/μL, 1.05×10^2^ copies/μL, 50 copies/μL, 10 copies/μL, 5 copies/μL, 1 copy/μL of *armA*-positive plasmid DNA, negative control (WHCDC-FY57), and blank control (deionized water) respectively. Lines 7–9 also represented negative signals and were therefore omitted from the **Figure 3C**.

To evaluate the specificity of MCDA-CRISPR/Cas12a for detecting the *armA* gene, genomic DNA from 22 *armA*-producing strains and 24 strains carrying a variety of resistant genes (*blaNDM-1*, *blaIMP-*4, *blaKPC-2*, *blaOXA-1*, *rmtB*, *qnrS*1, *blaTEM-1B* and *sul1*) were extracted. In addition, a plasmid carrying *rmtA* gene was added to the specificity panel. The real-time fluorescence results showed that only strains carrying *armA* gene were positive, while these non-*armA* species tested negative (**Figure 4**). BLAST analysis was performed to show the comparison of *armA* with the closest non-*armA* sequence and other 16S rRNA methylase genes. The data demonstrated that all other 16S rRNA methylase genes either showed no significant similarity to the *armA* or shared similarity only within a very small local region (query cover ≤ 11%), thereby providing strong bioinformatic evidence for the high specificity of the MCDA-CRISPR/Cas12a detection system for the *armA* gene (**Supplementary Table S4**). The above information indicated that the MCDA-CRISPR/Cas12a technology can specifically identify the *armA* gene.

**Figure 4:**
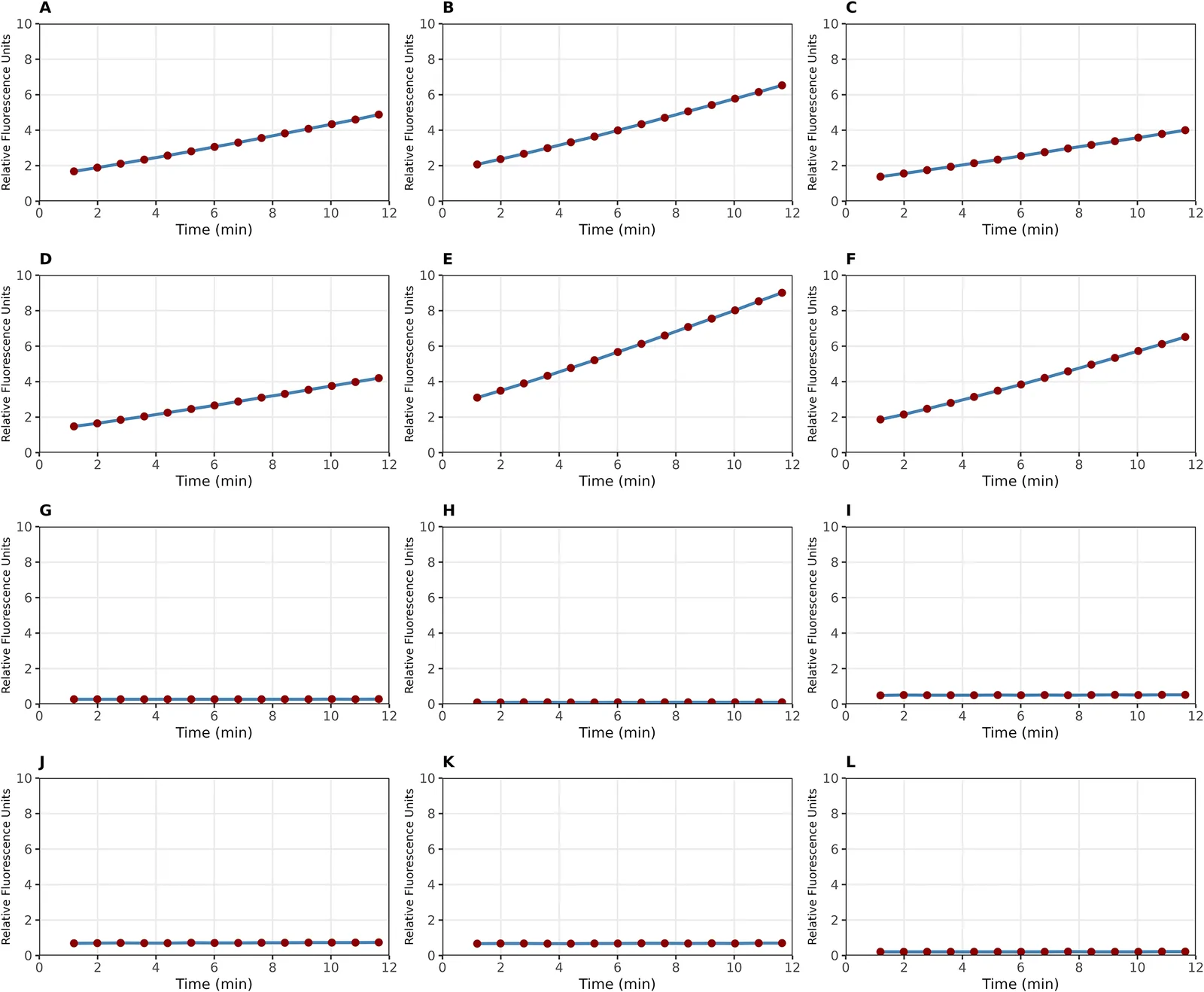
Specificity of *armA* MCDA-CRISPR/Cas12a assay. Graphs **(A–F)** *Enterobacter cloacae* FY85, *Enterobacter cloacae* PA40, *Escherichia coli* SY2, *Escherichia coli* YY110, *Klebsiella pneumoniae* YY44, *Klebsiella pneumoniae* YY50 separately (these isolates produced the *armA* enzyme). Graphs **(G–J)** *blaNDM-1*-carrying *Klebsiella pneumoniae* FY50, *blaOXA-1*-producing *Escherichia coli* HP85, *rmtB*-carrying *Shigella flexneri* HH13, *qnrs1*-carrying *Escherichia coli* SY10. Graph **(K)** was *rmtA*-positive plasmid. Graph **(L)** was deionized water.

### Clinical sample verification

Genomic DNA was extracted from 129 bacterial strains collected from eight hospitals between 2018 and 2024. Following the MCDA-CRISPR/Cas12a assay, the real-time fluorescence results demonstrated that only strains harboring the *armA* gene tested positive, while all non-*armA* strains tested negative (**Supplementary Table S5**). Compared with the gold standard of whole-genome sequencing, the MCDA-CRISPR/Cas12a assay accurately identified all 21 *armA*-positive strains and 108 *armA*-negative strains, showing 100% concordance with the sequencing results. It is important to emphasize that this validation was performed on cultured isolates rather than directly on primary clinical specimens. These findings demonstrate the excellent detection performance of the established method for identifying *armA*-positive bacterial isolates in routine microbiology laboratory settings.

### Comparison of the MCDA-CRISPR/Cas12a method with other detection techniques for *armA*

In the conventional PCR assay, a serial dilution of purified plasmid DNA harboring the *armA* gene were employed as template in standard PCR system to determine the sensitivity of PCR assay. Agarose gel electrophoresis analysis showed that the detection range of the PCR was from 1.05×10^8^ copies/μL to 1.05×10^3^ copies/μL (**Supplementary Figure S5**). Therefore, compared to conventional PCR method, MCDA-CRISPR/Cas12a technique is more sensitive and less time-consuming for *armA* detection.

Here, the analytical performance of the proposed MCDA-CRISPR/Cas12a method was compared with several previously reported techniques for the detection of *armA (*Hu et al., 2013; Nagasawa et al., 2014; Oshiro et al., 2015; Foudraine et al., 2021). As summarized in **Table 2**, the loop-mediated isothermal amplification (LAMP) assay showed a sensitivity of 100 copies and 100.00% specificity, making it 10-fold less sensitive than our method. The GeXP analyzer-based multiplex PCR assay demonstrated a comparable sensitivity of 10 copies but a considerably lower specificity of 85.71%, which may limit its practical reliability. For protein-based methods, LC-MS/MS reported a specificity of 99.49% without providing sensitivity data, whereas immunochromatography achieved a sensitivity of 12 ng of *armA* protein (approximately 10^6^ to 10^8^ gene copies) with 100.00% specificity. Notably, the sensitivity of immunochromatography is several orders of magnitude lower than that of the MCDA-CRISPR/Cas12a method when converted to copy number. Overall, the established method offers the highest sensitivity and excellent specificity, outperforming or matching all compared methods.

**Table 2:** Comparative analysis of the MCDA-CRISPR/Cas12a assay and other *armA* detection platforms.

Method	Sensitivity	Specificity
Targeted liquid chromatography-tandem mass spectrometry (Foudraine et al., 2021)	/^a^	99.49%
Immunochromatography (Jia et al., 2024)	12ng *armA* protein (10^6^~ 10^8^ copies)^b^	100.00%
Loop-mediated isothermal amplification assay (Wang et al., 2016)	10^2^ copies	100.00%
GeXP analyzer-based multiplex PCR assay (Hu et al., 2013)	10 copies	85.71%
Conventional PCR method (this study)	10^3^ copies	100.00%
MCDA-CRISPR/Cas12a assay (this study)	10 copies	100.00%

a ”/”, not reported.

b Values in parentheses indicate the estimated copy numbers derived from the detected *armA* protein quantity.

## Discussion

The *armA* gene, encoding a 16S rRNA methylase, plays a crucial role as a key determinant of high-level aminoglycoside resistance among clinically significant bacterial pathogens (Gondal et al., 2024). The gene encoding this resistance is typically located on plasmids, transposons, or a class of integrons (Zhang et al., 2008). Owing to its high mobility, it can be widely transferred and spread among bacterial strains, leading to an increasing number of Gram-negative bacteria developing high-level resistance to aminoglycoside antibiotics (Zhou et al., 2022). Thus, development of a rapid and reliable detection assay for *armA* gene will enhance the detection and monitoring of enzyme-producing strains, and also provide auxiliary guidance for the prevention and control of drug resistance issues in clinical, environmental, and livestock industries. In this study, we combined MCDA with CRISPR/Cas12a to create a detection platform for *armA*. The method is both fast and accurate, with good sensitivity. Operating at a constant temperature, the MCDA pre-amplifies the target sequence during the assay. This pre-amplification triggers the *trans*-cleavage activity of Cas12a, which then cleaves the MCDA products and ssDNA probes in the reaction. The final result can be easily determined by measuring the fluorescence intensity.

MCDA usually gives better specificity than other isothermal amplification methods such as Loop-mediated Isothermal Amplification (LAMP). That’s because it uses ten primers that can recognize up to different regions of the target sequence (Mao et al., 2025; Yuan et al., 2026). RPA has several limitations. It requires multiple proteins, including DNA polymerase, recombinase, and single-strand binding protein, whereas MCDA relies on a single enzyme, Bst DNA polymerase (Yuan et al., 2026). In addition, the reaction components of RPA cannot be pre-mixed in a single tube, the activator magnesium acetate must be added separately at the final step. RPA also suffers from non-specific amplification, which complicates result interpretation (Gong et al., 2023). MCDA is rarely troubled by these issues. Here, we compared fluorescence signal detection with colorimetric indication and gel electrophoresis after MCDA magnification, the experiment results showed that fluorescence detection demonstrated a high degree of accuracy and sensitivity. Colorimetric indication is based on observable color changes, while gel electrophoresis relies on band visualization. Both approaches become challenging at very low target concentrations (Wang et al., 2017). Additional, Lateral Flow Biosensor (LFB) has also been used in some studies for detecting MCDA products (Huang et al., 2023; Yuan et al., 2026). While LFB offers rapid results (within 1–2 minutes), it carries a risk of aerosol contamination when the reaction tube is opened (Gong et al., 2023).

The specificity of the newly established assay was validated using *armA*-positive and non-*armA* organisms, and the results were clear: all *armA*-positive isolates gave positive signals, while no signal was observed in any of the negative isolates, *rmtA* plasmid or the blank control. In addition, the combination of crRNA and primers ensured high specificity by precisely recognizing the *armA* sequence. These findings indicate that the MCDA-CRISPR/Cas12a assay is highly selective and can reliably detect the *armA* gene in bacterial samples. Notably, previous surveillance studies have shown that the prevalence of the *armA* gene among clinical isolates varies considerably across regions and bacterial species (Ma et al., 2009; Liu et al., 2025). Its relatively low abundance in unprocessed clinical specimens presents a significant technical challenge for direct detection methods. In standard clinical workflows, bacteria are first isolated from patient samples, followed by drug resistance testing. Consequently, a clinically relevant validation can be achieved by directly detecting the resistance gene in these isolated strains, which aligns well with real-world practice. A total of 129 carbapenemase-producing strains, previously confirmed by whole-genome sequencing, were collected from eight hospitals in Wuhan. The results obtained by the proposed method were fully consistent with the whole-genome sequencing data, providing strong evidence for the reliability of this method.

The assay designed in this study proved to be highly sensitive, achieving a detection limit as low as 10 copies/μL of *armA*-positive plasmid DNA. The detection limit of this method is comparable to that of other MCDA-CRISPR assays for pathogens, including *Brucella* genus, *B. melitensis*, and *human adenovirus (*Jia et al., 2025; Mao et al., 2025). Similarly, for the detection of resistance genes, the designed technique achieves a limit of detection equivalent to other established isothermal amplification-CRISPR/Cas approaches, including LAMP-CRISPR/Cas12a for the macrolide resistance gene *ermB* and RPA-CRISPR/Cas13a for the carbapenem resistance genes *blaKPC* and *blaNDM (*Cao et al., 2024; Mao et al., 2024). As described in **Table 2**, the proposed method exhibited 100-fold higher sensitivity than conventional PCR and 10-fold higher sensitivity than LAMP, while maintaining comparable specificity (100%). In specimens with low bacterial loads, such as blood or bronchoalveolar lavage fluid, or after antibiotic exposure, this difference may increase the likelihood of detection and reduce false-negative results. Although conventional PCR is the most frequently used method for gene diagnosis, the insensitivity and time-consuming defects make it not suit for on-site detection. The GeXP-based multiplex PCR method showed a similar sensitivity of 10 copies, whereas its specificity was considerably lower (85.71%). In contrast, protein-based methods such as targeted LC-MS/MS and immunochromatography exhibited high specificity (99.49% and 100%, respectively), but the latter required a greater abundance of target materials, making it several orders of magnitude less sensitive than our method. Collectively, the MCDA-CRISPR/Cas12a assay offers an optimal balance of ultra-high sensitivity and absolute specificity, positioning it as a promising tool for rapid and accurate detection of *armA* and other antimicrobial resistance determinants.

The MCDA-CRISPR/Cas12a assay developed in this study offers several advantages over conventional molecular methods, including rapid turnaround time (completable within 70 minutes), isothermal amplification (eliminating the need for thermal cyclers), and high sensitivity and specificity. However, a realistic assessment of its clinical utility must consider where this assay fits within existing diagnostic workflows. In its current form, the assay is best suited for use in reference or centralized clinical microbiology laboratories rather than as a true point-of-care test (POCT). After primary culture isolation, the assay can be performed on pure bacterial colonies to rapidly confirm *armA*-mediated aminoglycoside resistance. This would enable earlier targeted antibiotic therapy adjustments compared to conventional PCR or sequencing-based methods. The workflow, including DNA extraction (15 min), MCDA amplification (40 min), CRISPR/Cas12a cleavage (5 min), and fluorescence detection (10 min), is compatible with batch processing in laboratory settings.

Several practical challenges currently limit the deployment of this assay at the point of care. First, while MCDA operates at a constant temperature, it still requires a fluorescence detector, which may not be available in resource-limited settings. Second, the DNA extraction step, although rapid (15 min), requires centrifugation and pipetting steps that demand technical expertise and a minimally equipped workspace. Third, the CRISPR/Cas12a reagents require cold-chain storage, which may be challenging in field settings. Potential solutions to overcome these barriers include the development of integrated microfluidic devices that combine DNA extraction, amplification, and detection in a closed system, as well as the use of lyophilized reagents to eliminate cold-chain requirements.

## Conclusions

We have developed a simple, rapid, and accuracy MCDA-CRISPR/Cas12a assay for *armA* gene detection. With a limit of detection of 10 copies/μL of *armA*-positive plasmid DNA and 100% specificity as validated by our trial, this assay offers reliable performance. Therefore, the *armA*-MCDA-CRISPR/Cas12a assay established here holds great promise for significantly enhancing the detection efficiency of the target gene in real-world monitoring.

## Data Availability

The original contributions presented in the study are included in the article/**Supplementary Material**. Further inquiries can be directed to the corresponding author.
