# Supplementary material for: Highly sensitive and specific detection of aminoglycoside resistance methylase gene armA by multiple cross displacement amplification-CRISPR/Cas12a technique
Source: Front Cell Infect Microbiol. 2026 Oct 28;16:1947114. doi: 10.3389/fcimb.2026.1947114 (PMC13751484; doi:10.3389/fcimb.2026.1947114)
Supplement: Supplementary file 1 [file DataSheet1.docx]

**s**

**Supplementary Information**

**1. Supplementary Tables**

**Table S1.** Strains used in this study.

| **Genotype**  **^a^** | **Bacteria species** | **The source of strains^a^**  **^b^** | **No. of isolates** |
| --- | --- | --- | --- |
| *armA* | *Escherichia coli* | WHCDC | 2 |
|  | *Klebsiella pneumoniae* | WHCDC | 10 |
|  | *Pseudomonas aeruginosa* | WHCDC | 1 |
|  | *Enterobacter cloacae* | WHCDC | 9 |
| *blaKPC-2* | *Klebsiella pneumoniae* | WHCDC | 2 |
| *rmtB* | *Klebsiella pneumoniae* | WHCDC | 2 |
|  | *Escherichia coli* | WHCDC | 1 |
|  | *Shigella flexneri* | WHCDC | 1 |
|  | *Pseudomonas aeruginosa* | WHCDC | 1 |
| *blaTEM-1B* | *Klebsiella pneumoniae* | WHCDC | 1 |
| *blaNDM-1* | *Klebsiella pneumoniae* | WHCDC | 2 |
|  | *Enterobacter cloacae* | WHCDC | 3 |
| *blaIMP-4* | *Enterobacter cloacae* | WHCDC | 1 |
| *blaOXA-1* | *Escherichia coli* | WHCDC | 2 |
| *qnrS1* | *Escherichia coli* | WHCDC | 1 |
| *sul1* | *Pseudomonas aeruginosa* | WHCDC | 1 |
|  | *Escherichia coli* | WHCDC | 1 |
| Non^b^ | *Escherichia coli* | WHCDC | 1 |
|  | *Pseudomonas aeruginosa* | WHCDC | 2 |
|  | *Enterobacter aerogenes* | WHCDC | 1 |
|  | *Acinetobacter baumannii* | WHCDC | 1 |

^a^ WHCDC, Wuhan Center for Disease Control & Prevention. ^b^ Non, the stains did not carry above genes.

**Table S2.** Clinical sample information used in this study.

| **Number** | **Strain** | **Strain Source** | **Isolation date** | **Resistance gene *armA^a^*** | **Hospital Code^b^** |
| --- | --- | --- | --- | --- | --- |
| Ⅰ-17 | *Enterobacter cloacae* | Catheter tip | 30/Aug/2018 | - | Ⅰ |
| Ⅰ-18 | *Klebsiella pneumoniae* | Urine | 6/Sep/2018 | - | Ⅰ |
| Ⅰ-25 | *Klebsiella pneumoniae* | Sputum | 25/Sep/2018 | - | Ⅰ |
| Ⅰ-29 | *Escherichia coli* | Urine | 30/Oct/2018 | - | Ⅰ |
| Ⅰ-30 | *Klebsiella pneumoniae* | Sputum | 11/Nov/2018 | - | Ⅰ |
| Ⅰ-31 | *Klebsiella pneumoniae* | Bronchoalveolar lavage fluid | 13/Nov/2018 | - | Ⅰ |
| Ⅰ-36 | *Klebsiella pneumoniae* | Secretions | 20/Jan/2019 | - | Ⅰ |
| Ⅰ-41 | *Acinetobacter baumannii* | Catheter tip | 22/Feb/2019 | + | Ⅰ |
| Ⅰ-42 | *Escherichia coli* | Sputum | 28/Feb/2019 | - | Ⅰ |
| Ⅰ-44 | *Escherichia coli* | Catheter tip | 1/Mar/2019 | - | Ⅰ |
| Ⅰ-71 | *Acinetobacter baumannii* | Catheter tip | 26/Jun/2019 | - | Ⅰ |
| Ⅰ-73 | *Acinetobacter baumannii* | Sputum | 11/Jul/2019 | - | Ⅰ |
| Ⅰ-74 | *Klebsiella oxytoca* | Catheter tip | 12/Jul/2019 | - | Ⅰ |
| Ⅰ-83 | *Citrobacter freundii* | Catheter tip | 26/Oct/2019 | - | Ⅰ |
| Ⅰ-85 | *Enterobacter cloacae* | Sputum | 15/Nov/2019 | + | Ⅰ |
| Ⅰ-88 | *Acinetobacter pittii* | Sputum | 2/Dec/2019 | - | Ⅰ |
| Ⅰ-90 | *Acinetobacter pittii* | Bronchoalveolar lavage fluid | 9/Dec/2019 | - | Ⅰ |
| Ⅰ-92 | *Klebsiella pneumoniae* | Catheter tip | 21/Dec/2019 | - | Ⅰ |
| Ⅰ-97 | *Klebsiella pneumoniae* | Sputum | 1/Jan/2020 | - | Ⅰ |
| Ⅰ-139 | *Klebsiella aerogenes* | Sputum | 3/Apr/2022 | - | Ⅰ |
| Ⅰ-142 | *Klebsiella pneumoniae* | Urine | 8/Jul/2022 | - | Ⅰ |
| Ⅰ-160 | *Escherichia coli* | Urine | 17/Mar/2023 | - | Ⅰ |
| Ⅰ-188 | *Acinetobacter baumannii* | Catheter tip | 28/Feb/2024 | + | Ⅰ |
| Ⅰ-193 | *Acinetobacter baumannii* | Sputum | 19/May/2024 | + | Ⅰ |
| Ⅱ-41 | *Klebsiella pneumoniae* | Urine | 7/Aug/2019 | - | Ⅱ |
| Ⅱ-71 | *Klebsiella pneumoniae* | Sputum | 8/Oct/2019 | - | Ⅱ |
| Ⅱ-72 | *Acinetobacter baumannii* | Bronchoalveolar lavage fluid | 8/Oct/2019 | + | Ⅱ |
| Ⅱ-73 | *Klebsiella pneumoniae* | Urine | 10/Oct/2019 | - | Ⅱ |
| Ⅱ-84 | *Acinetobacter baumannii* | Sputum | 5/Dec/2019 | + | Ⅱ |
| Ⅱ-93 | *Klebsiella pneumoniae* | Secretions | 2/Apr/2021 | - | Ⅱ |
| Ⅱ-95 | *Escherichia coli* | Blood | 9/Apr/2021 | - | Ⅱ |
| Ⅱ-135 | *Shigella flexneri* | Pus | 3/Jun/2023 | - | Ⅱ |
| Ⅱ-139 | *Klebsiella pneumoniae* | Sputum | 10/Sep/2023 | - | Ⅱ |
| Ⅱ-140 | *Acinetobacter bereziniae* | Sputum | 1/Aug/2023 | - | Ⅱ |
| Ⅱ-164 | *Escherichia coli* | Urine | 12/Aug/2024 | - | Ⅱ |
| Ⅲ-1 | *Pseudomonas aeruginosa* | Urine | 16/Jul/2018 | - | Ⅲ |
| Ⅲ-2 | *Enterobacter cloacae* | Urine | 15/Jul/2018 | - | Ⅲ |
| Ⅲ-10 | *Acinetobacter baumannii* | Urine | 24/Aug/2018 | + | Ⅲ |
| Ⅲ-16 | *Enterobacter cloacae* | Secretions | 23/Sep/2018 | - | Ⅲ |
| Ⅲ-40 | *Klebsiella pneumoniae* | Blood | 30/Jan/2019 | - | Ⅲ |
| Ⅲ-41 | *Acinetobacter baumannii* | Urine | 31/Jan/2019 | + | Ⅲ |
| Ⅲ-94 | *Klebsiella aerogenes* | Sputum | 31/Aug/2020 | - | Ⅲ |
| Ⅲ-96 | *Enterobacter cloacae* | Sputum | 11/Sep/2020 | - | Ⅲ |
| Ⅲ-258 | *Klebsiella michiganensis* | Urine | 3/May/2022 | - | Ⅲ |
| Ⅲ-260 | *Enterobacter hormaechei* | Sputum | 6/May/2022 | - | Ⅲ |
| Ⅲ-261 | *Acinetobacter baumannii* | Sputum | 7/May/2022 | + | Ⅲ |
| Ⅲ-279 | *Escherichia coli* | Secretions | 3/Dec/2023 | - | Ⅲ |
| Ⅲ-280 | *Klebsiella quasipneumoniae* | Sputum | 20/Dec/2023 | + | Ⅲ |
| Ⅲ-281 | *Escherichia coli* | Secretions | 11/Jun/2024 | - | Ⅲ |
| Ⅳ-3 | *Escherichia coli* | Urine | 26/Jul/2018 | - | Ⅳ |
| Ⅳ-6 | *Pseudomonas aeruginosa* | Sputum | 8/Jul/2018 | - | Ⅳ |
| Ⅳ-12 | *Klebsiella pneumoniae* | Secretions | 18/Jul/2018 | + | Ⅳ |
| Ⅳ-15 | *Pseudomonas aeruginosa* | Sputum | 16/Aug/2018 | - | Ⅳ |
| Ⅳ-16 | *Klebsiella pneumoniae* | Urine | 21/Aug/2018 | - | Ⅳ |
| Ⅳ-28 | *Citrobacter freundii* | Bronchoalveolar lavage fluid | 8/Jan/2019 | - | Ⅳ |
| Ⅳ-37 | *Acinetobacter baumannii* | Secretions | 19/Feb/2019 | + | Ⅳ |
| Ⅳ-40 | *Enterobacter cloacae* | Secretions | 29/Mar/2019 | + | Ⅳ |
| Ⅳ-42 | *Klebsiella pneumoniae* | Sputum | 27/Apr/2019 | - | Ⅳ |
| Ⅳ-53 | *Klebsiella pneumoniae* | Urine | 7/Jun/2019 | - | Ⅳ |
| Ⅳ-54 | *Citrobacter youngae* | Urine | 15/Jun/2019 | - | Ⅳ |
| Ⅳ-56 | *Escherichia coli* | Urine | 30/Jul/2019 | - | Ⅳ |
| Ⅳ-100 | *Acinetobacter baumannii* | Urine | 18/Feb/2022 | + | Ⅳ |
| Ⅳ-101 | *Escherichia coli* | Blood | 24/Feb/2022 | - | Ⅳ |
| Ⅳ-104 | *Citrobacter portucalensis* | Blood | 20/Mar/2022 | - | Ⅳ |
| Ⅳ-106 | *Klebsiella pneumoniae* | Urine | 31/Mar/2022 | - | Ⅳ |
| Ⅳ-112 | *Klebsiella pneumoniae* | Sputum | 12/Jun/2022 | - | Ⅳ |
| Ⅳ-119 | *Klebsiella pneumoniae* | Sputum | 25/Nov/2023 | - | Ⅳ |
| Ⅳ-120 | *Enterobacter cloacae* | Urine | 26/Nov/2023 | - | Ⅳ |
| Ⅳ-122 | *Klebsiella pneumoniae* | Urine | 27/Nov/2023 | - | Ⅳ |
| Ⅳ-124 | *Klebsiella michiganensis* | Sputum | 28/Nov/2023 | - | Ⅳ |
| Ⅳ-128 | *Klebsiella pneumoniae* | Sputum | 29/Nov/2023 | - | Ⅳ |
| Ⅳ-132 | *Klebsiella pneumoniae* | Sputum | 30/Nov/2023 | - | Ⅳ |
| Ⅳ-137 | *Escherichia coli* | Wound drainage | 1/Dec/2023 | - | Ⅳ |
| Ⅳ-139 | *Enterobacter hormaechei* | Blood | 2/Dec/2023 | - | Ⅳ |
| Ⅳ-144 | *Escherichia coli* | Bile | 4/Sep/2024 | - | Ⅳ |
| Ⅳ-148 | *Escherichia coli* | Wound drainage | 18/Sep/2024 | - | Ⅳ |
| Ⅴ-7 | *Klebsiella pneumoniae* | Sputum | 3/Sep/2018 | - | Ⅴ |
| Ⅴ-31 | *Klebsiella pneumoniae* | Sputum | 21/Jun/2019 | - | Ⅴ |
| Ⅴ-32 | *Klebsiella pneumoniae* | Sputum | 1/Jul/2019 | - | Ⅴ |
| Ⅴ-35 | *Acinetobacter baumannii* | Urine | 2/Sep/2019 | - | Ⅴ |
| Ⅴ-40 | *Acinetobacter baumannii* | Sputum | 25/Oct/2019 | + | Ⅴ |
| Ⅴ-43 | *Klebsiella pneumoniae* | Sputum | 4/Nov/2019 | - | Ⅴ |
| Ⅴ-56 | *Klebsiella pneumoniae* | Sputum | 24/Jan/2022 | - | Ⅴ |
| Ⅴ-59 | *Klebsiella pneumoniae* | Urine | 17/Jun/2022 | - | Ⅴ |
| Ⅴ-60 | *Acinetobacter baumannii* | Blood | 30/Jul/2022 | + | Ⅴ |
| Ⅴ-71 | *Escherichia coli* | Urine | 16/Oct/2023 | - | Ⅴ |
| Ⅵ-21 | *Klebsiella pneumoniae* | Urine | 14/Oct/2018 | - | Ⅵ |
| Ⅵ-30 | *Klebsiella pneumoniae* | Bronchoalveolar lavage fluid | 10/Dec/2018 | - | Ⅵ |
| Ⅵ-31 | *Klebsiella pneumoniae* | Bronchoalveolar lavage fluid | 15/Jan/2019 | - | Ⅵ |
| Ⅵ-35 | *Klebsiella pneumoniae* | Sputum | 14/Jan/2019 | - | Ⅵ |
| Ⅵ-69 | *Klebsiella pneumoniae* | Sputum | 23/Feb/2019 | - | Ⅵ |
| Ⅵ-70 | *Klebsiella pneumoniae* | Blood | 20/Feb/2019 | - | Ⅵ |
| Ⅵ-73 | *Klebsiella pneumoniae* | Sputum | 12/Mar/2019 | - | Ⅵ |
| Ⅵ-75 | *Proteus mirabilis* | Urine | 1/Apr/2019 | - | Ⅵ |
| Ⅵ-76 | *Enterobacter cloacae* | Bronchoalveolar lavage fluid | 8/Apr/2019 | + | Ⅵ |
| Ⅵ-79 | *Escherichia coli* | Sputum | 28/May/2019 | - | Ⅵ |
| Ⅵ-101 | *Klebsiella pneumoniae* | Sputum | 30/Aug/2019 | - | Ⅵ |
| Ⅵ-102 | *Escherichia coli* | Blood | 30/Aug/2019 | - | Ⅵ |
| Ⅵ-105 | *Klebsiella pneumoniae* | Sputum | 3/Sep/2019 | - | Ⅵ |
| Ⅵ-110 | *Escherichia coli* | Secretions | 31/Oct/2019 | + | Ⅵ |
| Ⅵ-111 | *Klebsiella pneumoniae* | Sputum | 5/Nov/2019 | - | Ⅵ |
| Ⅵ-139 | *Shigella flexneri* | Bile | 15/Jul/2021 | - | Ⅵ |
| Ⅵ-173 | *Klebsiella pneumoniae* | Sputum | 12/Nov/2021 | - | Ⅵ |
| Ⅵ-181 | *Acinetobacter johnsonii* | Urine | 13/Dec/2021 | - | Ⅵ |
| Ⅵ-188 | *Enterobacter cloacae* | Secretions | 21/Jan/2022 | + | Ⅵ |
| Ⅵ-193 | *Klebsiella pneumoniae* | Bronchoalveolar lavage fluid | 7/Jan/2022 | - | Ⅵ |
| Ⅵ-194 | *Klebsiella pneumoniae* | Urine | 17/Feb/2022 | - | Ⅵ |
| Ⅵ-207 | *Enterobacter cloacae* | Bronchoalveolar lavage fluid | 13/May/2022 | + | Ⅵ |
| Ⅵ-212 | *Klebsiella pneumoniae* | Sputum | 3/Jun/2022 | - | Ⅵ |
| Ⅵ-222 | *Klebsiella pneumoniae* | Urine | 28/Jun/2022 | - | Ⅵ |
| Ⅵ-223 | *Enterobacter hormaechei* | Urine | 28/Jun/2022 | - | Ⅵ |
| Ⅵ-230 | *Klebsiella pneumoniae* | Sputum | 23/Jul/2022 | - | Ⅵ |
| Ⅵ-231 | *Enterobacter hormaechei* | Sputum | 22/Jul/2022 | - | Ⅵ |
| Ⅵ-232 | *Klebsiella pneumoniae* | Bronchoalveolar lavage fluid | 9/Aug/2022 | - | Ⅵ |
| Ⅵ-247 | *Enterobacter hormaechei* | Sputum | 6/Sep/2022 | - | Ⅵ |
| Ⅵ-296 | *Escherichia coli* | Blood | 25/Aug/2023 | - | Ⅵ |
| Ⅵ-297 | *Klebsiella pneumoniae* | Sputum | 31/Aug/2023 | - | Ⅵ |
| Ⅵ-306 | *Enterobacter cloacae* | Sputum | 28/Oct/2023 | - | Ⅵ |
| Ⅵ-307 | *Enterobacter hormaechei* | Urine | 31/Oct/2023 | - | Ⅵ |
| Ⅵ-323 | *Enterobacter cloacae* | Sputum | 1/Jul/2024 | - | Ⅵ |
| Ⅶ-12 | *Escherichia coli* | Urine | 28/Mar/2022 | - | Ⅶ |
| Ⅶ-13 | *Shigella flexneri* | Urine | 12/Apr/2022 | - | Ⅶ |
| Ⅷ-3 | *Klebsiella pneumoniae* | Throat swab | 25/Nov/2020 | - | Ⅷ |
| Ⅷ-4 | *Klebsiella pneumoniae* | Anal swab | 26/Nov/2020 | - | Ⅷ |
| Ⅷ-18 | *Escherichia coli* | Urine | 16/Jan/2021 | - | Ⅷ |
| Ⅷ-19 | *Klebsiella pneumoniae* | Anal swab | 18/Jan/2021 | - | Ⅷ |
| Ⅷ-29 | *Enterobacter hormaechei* | Sputum | 24/Nov/2021 | - | Ⅷ |
| Ⅷ-32 | *Klebsiella pneumoniae* | Secretions | 25/Nov/2021 | + | Ⅷ |
| Ⅷ-33 | *Klebsiella pneumoniae* | Sputum | 9/Jan/2022 | - | Ⅷ |

^a^“+” represents positive, and “-” represents negative. ^b^ Codes Ⅰ-Ⅷ represent the eight participating hospitals in Wuhan.

**Table S3.** Detection rate, reproducibility, and signal-to-noise ratio of the MCDA-CRISPR/Cas12a assay at different plasmid DNA concentrations.

| Concentration (copies/μL) | Detection Rate | Mean ± SD^a^ | CV^b^ | SNR^c^ |
| --- | --- | --- | --- | --- |
| 1.05×10^4^ | 7/7 (100%) | 14.69 ± 2.21 | 15.04% | 116.42 |
| 1.05×10^3^ | 7/7 (100%) | 12.63 ± 2.41 | 19.08% | 99.25 |
| 1.05×10^2^ | 7/7 (100%) | 18.12 ± 3.58 | 19.76% | 145.00 |
| 50 | 7/7 (100%) | 14.31 ± 2.83 | 19.78% | 113.25 |
| 10 | 7/7 (100%) | 9.46 ± 2.08 | 21.99% | 72.83 |
| 5 | 0/7 (0%) | 0.78 ± 0.11 | 14.10% | 0.50 |
| 1 | 0/7 (0%) | 0.91 ± 0.07 | 7.69% | 1.58 |
| negative control | 0/7 (0%) | 0.77 ± 0.06 | 7.79% | 0.42 |
| blank control (NTC^d^) | 0/7 (0%) | 0.72 ± 0.12 | 16.67% | /^e^ |

^a^ SD, Standard Deviation; ^b^ CV, Coefficient of Variation; ^c^ SNR, Signal-to-Noise Ratio; ^d^ NTC, No-Template Control; ^e^ /, Not applicable.

**Table S4.** Comparison of *armA* with the closest non-*armA* sequence and other 16S rRNA methylase genes.

| **Target Gene** | **GenBank Accession** | **Sequence Identity to *armA*** | **Query Cover** |
| --- | --- | --- | --- |
| *armA* | KU984333.1 | 100% | 100% |
| Unknown  sequence^a^ | CP096742.1 | 100% | 9% |
| *rmtA* | AB083212.2 | No significant similarity | / |
| *rmtB* | FJ556899.1 | No significant similarity | / |
| *rmtC* | KJ476818.1 | 74.60% | 11% |
| *rmtD* | EU269034.2 | No significant similarity | / |
| *rmtE* | KT428293.1 | 92.31% | 9% |
| *rmtF* | JQ955744.1 | No significant similarity | / |
| *rmtG* | JX486113.1 | No significant similarity | / |
| *rmtH* | KC544262.1 | No significant similarity | / |
| *npmA* | AB261016.2 | No significant similarity | / |

^a^ The closest non-*armA* sequence was identified as an "Unknown sequence".

**Table S5.** Comparison of the MCDA-CRISPR/Cas12a assay with whole-genome sequencing for the analysis of clinical strains.

| MCDA-CRISPR/Cas12a assay | Whole-genome sequencing | | Total |  |
| --- | --- | --- | --- | --- |
|  | Positive | Negative |  |  |
| Positive | 21 | 0 | 21 | |
| Negative | 0 | 108 | 108 | |
| Total | 21 | 108 | 129 | |

**2. Supplementary Figures**

**Figure S1.** Sequence alignment of the *armA* target regions across different bacterial species.

**Figure S2. Optimal temperature for MCDA reaction.** The MCDA products of *armA*-positive plasmid DNA with a concentration of 1.05×10^5^ copies/μL in different temperatures were analyzed using electrophoresis experiment. Lanes 1-8, amplicons electrophoretogram at 60 to 67°C with 1°C increments. Lanes 9-10, negative control (WHCDC-FY57) and blank control (deionized water).

**Figure S3**. **Representative kinetic fluorescence curves for the sensitivity analysis of the MCDA-CRISPR/Cas12a assay**. Lines 1-6 represent serial dilutions of the *armA* plasmid from high to low concentrations (1.05×10^4^, 1.05×10^3^, 1.05×10^2^, 50, 10, and 5 copies/μL). The curves for 1 copy/μL, negative control and blank control were not displayed because they partially overlapped with that of 5 copies/μL, thereby improving the readability of the figure.

**Figure S4. Fluorescence curves for all replicates at the LoD (10 copies/μL). (A)** experiment 1; **(B)** experiments 2-7.

**Figure S5. Sensitivity of conventional PCR for *armA* detection.** The PCR products of *armA*-positive plasmid DNA were analyzed using electrophoresis experiment. Lanes 1-13 were 1.05×10^8^, 1.05×10^7^, 1.05×10^6^, 1.05×10^5^, 1.05×10^4^, 1.05×10^3^, 1.05×10^2^, 50, 10, 5, 1 copy/μL *armA*-positive plasmid DNA, negative control (WHCDC-FY57), and blank control (deionized water), respectively.
